# Therapeutic potentials and modulatory mechanisms of fatty acids in bone

**DOI:** 10.1111/cpr.12735

**Published:** 2019-12-04

**Authors:** Minyue Bao, Kaiwen Zhang, Yangyini Wei, Weihan Hua, Yanzi Gao, Xin Li, Ling Ye

**Affiliations:** ^1^ State Key Laboratory of Oral Diseases National Clinical Research Center for Oral Diseases West China Hospital of Stomatology Sichuan University Chengdu China; ^2^ State Key Laboratory of Oral Diseases National Clinical Research Center for Oral Diseases Department of Cariology and Endodontics West China Hospital of Stomatology Sichuan University Chengdu China

**Keywords:** bone diseases, bone homeostasis, bone metabolism, fatty acids

## Abstract

Bone metabolism is a lifelong process that includes bone formation and resorption. Osteoblasts and osteoclasts are the predominant cell types associated with bone metabolism, which is facilitated by other cells such as bone marrow mesenchymal stem cells (BMMSCs), osteocytes and chondrocytes. As an important component in our daily diet, fatty acids are mainly categorized as long‐chain fatty acids including polyunsaturated fatty acids (LCPUFAs), monounsaturated fatty acids (LCMUFAs), saturated fatty acids (LCSFAs), medium‐/short‐chain fatty acids (MCFAs/SCFAs) as well as their metabolites. Fatty acids are closely associated with bone metabolism and associated bone disorders. In this review, we summarized the important roles and potential therapeutic implications of fatty acids in multiple bone disorders, reviewed the diverse range of critical effects displayed by fatty acids on bone metabolism, and elucidated their modulatory roles and mechanisms on specific bone cell types. The evidence supporting close implications of fatty acids in bone metabolism and disorders suggests fatty acids as potential therapeutic and nutritional agents for the treatment and prevention of metabolic bone diseases.

## 1 INTRODUCTION

**Figure 1:**
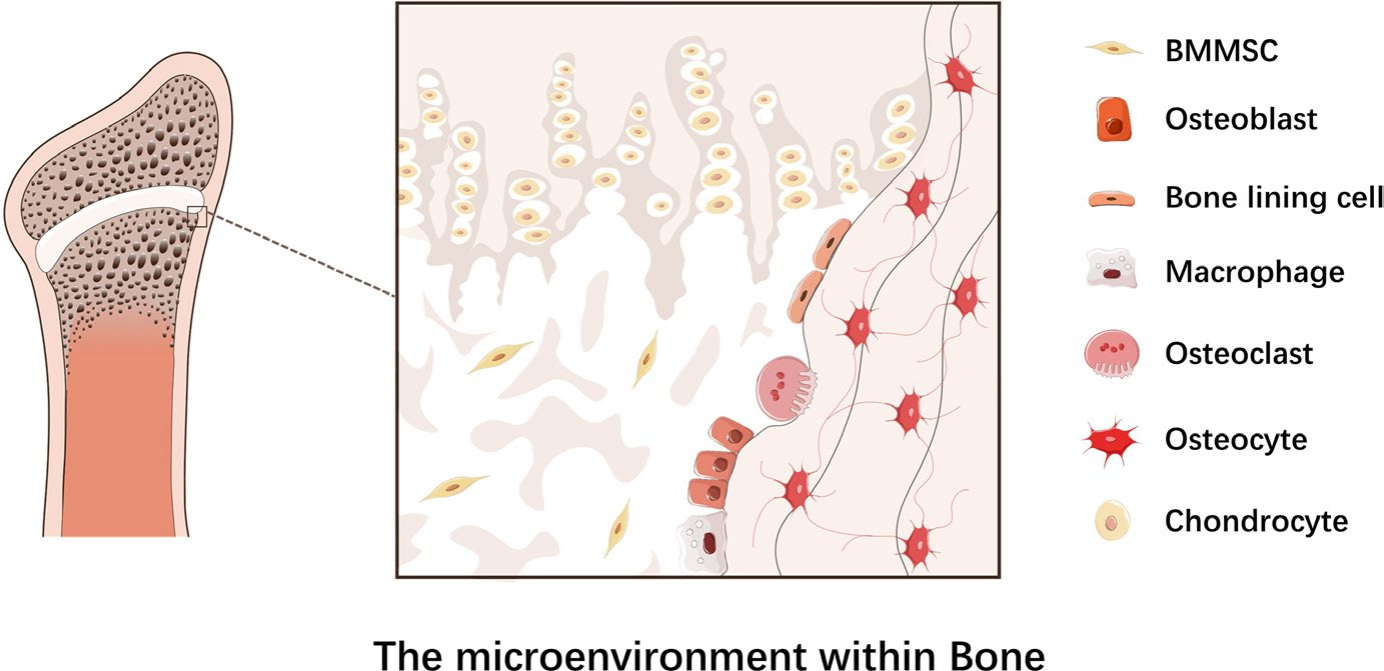
The microenvironment within bone. This figure displays the composition of bone microenvironment at cellular level. BMMSCs are multipotent cells capable of differentiating into multiple cell types such as osteoblasts. Osteoblasts are mononuclear cells responsible for bone formation. Bone lining cells are flat‐shaped cells located along the bony surfaces, maintaining their proliferative ability into other osteogenic cells. Macrophages are common precursors of osteoclasts and bone marrow‐resident macrophages in bone tissue. Osteoclasts are multinucleated giant cells with bone resorptive activity. Osteocytes are cells reside in bone lacunae and support bone structure, also with capacity to regulate the activities of both osteoclasts and osteoblasts. Chondrocytes are the main cartilage cell type existing in cartilaginous interstitium and cartilage lacuna. All these cell types existing in microenvironment within bone are implicated in bone homeostasis and thereby bone diseases

Bone metabolism including osteoclasts‐mediated bone resorption and osteoblasts‐mediated bone formation is a lifelong process occurring within cancellous as well as cortical bones. Bone resorption starts with recruitment of osteoclasts to mineralized bone tissues and leads to acidification of extracellular microenvironment. Osteoclasts dissolve hydroxyapatite mineral crystals by producing hydrogen ions and digesting organic bone matrix via synthesis of hydrolytic enzymes, both resulting in calcium transfer from bone tissue into blood.^1^, ^2^ Bone formation is initiated by bone marrow mesenchymal stem cells (BMMSCs) migrating from vascular channels circulation to bone surface. Osteoblasts deposit organic bone matrix and regulate its mineralization and eventually differentiate into osteocytes that are embedded in the cavities of mineralized matrix.^2^, ^3^ In addition to osteoclasts, BMMSCs and osteoblasts, other bone cell types participating in bone metabolism include macrophages, surface bone‐lining cells, chondrocytes as well as osteocytes (Figure 1).^4^, ^5^, ^6^, ^7^, ^8^, ^9^, ^10^

Accumulating evidence has established essential roles of fatty acids in bone metabolism 11 (Table 1). Categorization of fatty acids involved in bone metabolism has been reviewed by Natalia S. Harasymowicz *et al* 12 In general, ω‐3 long‐chain polyunsaturated fatty acids (LCPUFAs) are a group of well‐known fatty acids obtained from diet and supplemented via in vivo synthesis,^13^ and eicosapentaenoic acid (EPA), alpha‐linolenic acid (ALA) and docosahexaenoic acid (DHA) are the three major representatives of ω‐3 LCPUFAs. ω‐3 LCPUFAs could mediate bone metabolism *via* processes including lipid oxidation, calcium absorption and prostaglandin synthesis,^14^ and they can exert beneficial effects on bone remodelling by inhibiting osteoclast activity and enhancing osteoblast activity.^15^ Several studies have investigated the therapeutic properties of ω‐3 LCPUFAs. By promoting bone formation, ω‐3 LCPUFAs significantly affect peak bone mass,^16^ increase bone calcium levels as well as bone mineral content (BMC) and density.^17^, ^18^, ^19^, ^20^ Therefore, they represent a non‐pharmacological strategy for preventing bone loss and accelerating fracture healing 21 and thus to reduce risks of osteoporosis and rheumatoid arthritis.^16^, ^22^, ^23^ In addition, ingestion of ω‐3 LCPUFAs eliminates adriamycin‐ or cyclophosphamide‐induced toxicity in bone marrow and bone tissues, suggesting potential roles of ω‐3 LCPUFAs in combating side effects of specific bone‐targeted drugs.^24^

**Table 1:** Overview of fatty acids involved in specific bone cell types and bone diseases

Fatty acid	Effects on bone metabolism	Molecular formula	Structural formula	Class	Targeted cell	Receptor	Pathway	Relevant disease	References
Eicosapentaenoic acid (EPA)	Promote bone formation	C_20_H_30_O_2_	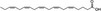	ω‐3 LCPUFAs	Osteoblasts Osteoclasts BMMSCs Chondrocytes	PPAR‐γ PTH1R GPR120 GPR40	MAPK mTOR	Rheumatoid arthritis; Periodontitis; Osteocarcinoma	181‐184, 205
Docosahexaenoic acid (DHA)	Promote bone formation; Inhibit bone resorption	C_22_H_32_O_2_	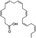	ω‐3 LCPUFAs	Osteoblasts Osteoclasts BMMSCs Chondrocytes	PPAR‐γ PTH1R TLR4 GPR120 GPR40	MAPK NF‐kB	Rheumatoid arthritis; Periodontitis; Bone fracture; Osteocarcinoma	181‐184, 205
a‐Linolenic Acid (ALA)	Inhibit bone resorption	C_18_H_30_O_2_	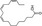	ω‐6 LCPUFAs	Osteoclasts Chondrocytes	GPR40	MAPK NF‐kB	—	181
Arachidonic Acid (AA)	Inhibit bone resorption	C_20_H_32_O_2_	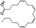	ω‐6 LCPUFAs	Osteoclasts Chondrocytes	TLR4	MAPK NF‐kB	Rheumatoid arthritis; Bone fracture	181‐184, 205
Myristic acid(MA)	Inhibit bone resorption	C_14_H_26_O_2_	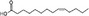	ω‐5 LCMUFAs	Osteoclasts	GPR40	RANKL	Osteolysis; Osteoporosis	25, 26
Palmitoleic acid(PLA)	Inhibit bone resorption	C_16_H_30_O_2_	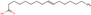	ω‐7 LCMUFAs	Osteoclasts	GPR40	NF‐kB MAPK	Rheumatoid arthritis; Osteoporosis; Osteosclerosis	166
Oleic acid (OA)	Inhibit bone resorption	C_18_H_34_O_2_	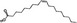	ω‐9 LCMUFAs	Osteoclasts BMMSCs	GPR40	NF‐kB MAPK	Bone healing; Osteoporosis; Periodontitis	168, 202
Palmitic acid (PA)	Enhance bone resorption; Inhibit bone formation	C_16_H_32_O_2_	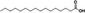	LCSFAs	Osteoclasts, Osteoblasts BMMSCs Osteocytes Chondrocytes	TLR4 PPAR‐γ	MAPK, mTOR BMP NF‐kB	Osteoarthritis; Bone loss; Periodontitis	105, 180
Stearic acid(SA)	Enhance bone resorption; Inhibit bone formation	C_18_H_36_O_2_	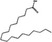	LCSFAs	Chondrocytes	—	NF‐kB	—	
Capric acid(CA)	Inhibit bone resorption	C_10_H_20_O_2_	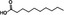	MCFAs	Osteoclasts	GPR120 GPR40 GPR84	MAPK, NF‐kB	Osteoporosis; Rheumatoid arthritis	214
Short chain FAs					Osteoclasts Osteoblasts Chondrocytes	GPR41 GPR43 GPR109		Inflammatory bone loss; Arthritis; Osteoporosis	29, 64
ResolvinE1 (RvE1)	Promote bone formation; Inhibit bone resorption	C_20_H_30_O_5_	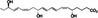	EPA metabolites	Osteoblasts Osteoclasts	ChemR23	NF‐κB, MAPK	Periodontitis	151
Lipoxin A4 (LXA4)	Inhibit bone resorption	C_20_H_32_O_5_	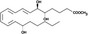	AA metabolites	Osteoclasts	FPR2/ALX	NF‐κB, MAPK mTOR	—	215
Prostaglandin E2 (PGE2)	Promote bone formation; Enhance bone resorption	C_20_H_32_O_5_	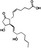	AA metabolites	Osteoblasts Osteoclasts Osteocytes Chondrocytes	EP_2_, EP_4_	RANKL	Periodontitis; Bone fracture	276

Long‐chain monounsaturated fatty acids (LCMUFAs) such as ω‐5, ω‐7 and ω‐9 categories are commonly recognized as potential agents against osteoporosis and other osteolytic diseases. They promote bone formation and inhibit bone degeneration and thereby facilitate bone metabolism. By contrast, long‐chain saturated fatty acids (LCSFAs) might negatively affect bone metabolism. Intake of common dietary SFAs such as lauric acid (LA, C12:0), myristic acid (MA, C14:0), palmitic acid (PA, C16:0) or stearic acid (SA, C18:0) might initiate inflammatory osteoarthritis and obesity.^25^, ^26^, ^27^ Moreover, medium‐chain fatty acids (MCFAs) such as capric acid (CA) have been reported to suppress osteoclastogenesis and thereby alleviate bone resorption. Short‐chain fatty acids (SCFAs)^28^, ^29^, ^30^ including acetate, butyrate and propionate have been suggested to inhibit bone resorption and combat inflammation. As a result, SCFAs are promising in the prevention of inflammatory bone loss and arthritis. Furthermore, fatty acid derivatives such as lipoxin A_4_ (LXA_4_) and resolvin E1 (RvE1) have also been involved in bone resorption attenuation. Therefore, considering large quantities of fatty acids in our daily diets, it is worthwhile to understand influences of fatty acids on bone metabolism and the underlying mechanisms, for further exploring their beneficial therapeutic applications in a wide variety of metabolic bone disorders.

## 2 IMPLICATIONS OF FATTY ACIDS IN BONE DISEASES

### 2.1 Periodontitis

Periodontitis is a chronic bacterial infection disease characterized by primary gingival and extended alveolar bone inflammation, accompanied by periodontal tissue damage 31, 32 with connective tissue degradation and even tooth loss.^33^ Consistent links between fatty acids and periodontitis have been established by evidence derived from animal and human subjects. Investigations in animal models from different groups such as Bendyk *et al* 34 and Azuma *et al* 35, 36 come to conclusions that tissue levels of ω‐3 LCPUFAs is inversely associated with periodontic alveolar bone loss,^34^ and ω‐3 LCPUFAs EPA metabolite RvE1 is also established to enhance bone formation and reduce bone resorption in rabbit periodontitis models.^37^ As for the anti‐inflammatory effects, LCMUFAs oleic acid (OA) exhibits anti‐inflammatory potentials to decrease alveolar bone loss, while LCSFAs PA shows contrary effects with elevated tumour necrosis factor α (TNF‐α) levels in obesity mice models,^38^ suggesting that the anti‐inflammatory potentials of fatty acids in periodontitis might be varied based on specific fatty acids types. In human subjects, increased concentrations of specific SCFAs (lactic acid, propionic acid, butyric acid, isovaleric acid) have been found in the gingival fluid of periodontitis patients, demonstrating a possible association between SCFAs and inflammatory alveolar bone loss.^39^, ^40^ Moreover, a preliminary clinical study by El‐Sharkawy *et al* suggests that dietary supplementation of ω‐3 LCPUFAs might have therapeutic values against periodontitis.^41^, ^42^, ^43^, ^44^, ^45^ However, there are also clinical investigations report that benefits of dietary ω‐3 LCPUFAs might not be applied to periodontitis prevention and treatment.^44^, ^46^

In mechanism, fatty acids might exert effects on periodontitis pathogenesis and intervention via direct and indirect mechanisms. Fatty acids could directly affect periodontitis‐associated bone destruction. LCSFAs such as PA could trigger *P gingivalis*‐induced alveolar bone loss directly.^31^ In benefit, EPA metabolite RvE1 could target BLT1 receptors in osteoclasts to inhibit osteoclast fusion and maturation, and RvE1 can induce the release of osteoprotegerin (OPG) to antagonize the proresorptive role of osteoclast‐stimulating receptor activator of nuclear kappa‐β ligand (RANKL), and thus facilitates the prevention of alveolar bone loss and enhances periodontal bone regeneration in periodontitis patients.^47^ The indirect effects of fatty acids in periodontitis are mainly through inflammatory response. Studies have shown that LCSFAs (such as PA) at high levels in plasma may facilitate *P gingivalis*‐induced chemokine production in human gingival fibroblasts and further promote inflammatory response in periodontium.^31^ PA‐induced chemokine secretion in human gingival fibroblasts could be inhibited by LCPUFAs (such as DHA), and such effects presumably involving the suppression of toll‐like receptor (TLR) dimerization as well as nuclear factor‐kappa B (NF‐κB) activation.^48^ In addition to exert effects on chemokine, fatty acids such as RvE1 could also act on inflammatory cells by enhancing the migration of monocytes and neutrophils and promoting the clearance of apoptotic neutrophils to enhance pro‐inflammatory response.^31^ Last but not the least, ω‐3 fatty acids such as DHA and EPA exhibit extensive antibacterial effects against putative periodontal pathogens including *F nucleatum* and *P gingivalis*, and SCFA butyrate derived from anaerobic bacterial metabolism could inhibit the differentiation of gingival fibroblasts to promote chronic periodontitis.^49^ Given that refractory periodontitis significantly decreases the life quality of patients, studies investigating the interaction between fatty acids and periodontitis are required to develop novel intervention strategies.

### 2.2 Osteoporosis

Osteoporosis, marked by low bone mineral density (BMD) and deteriorated bone tissue microarchitecture, contributes to a high incidence of bone fracture on average up to 50% of women > 50 years.^50^ Osteoporosis is mainly caused by excessive bone resorption resulting from imbalance between overactive osteoclasts and inactive osteoblasts.^51^, ^52^ Hence, inhibiting bone resorption or promoting bone formation are promising strategies for osteoporosis prevention and treatment.^53^ It has been well acknowledged that osteoporosis is associated with levels of fatty acid in bone microenvironment.^54^ As reviewed earlier by Salari *et al*,^55^ investigations conducted in humans have shown inconsistent correlations between fatty acids and osteoporosis, while studies in animal models have confirmed that supplementation of ω‐3 LCPUFAs alleviates osteoporosis by suppressing bone breakdown, promoting calcium absorption from diet, reducing prostaglandin E2 (PGE2) production and increasing skeletal calcium.^56^ In mechanism, ω‐6 LCPUFAs intake results in a high ratio of ω‐6 versus ω‐3 LCPUFAs, and thus facilitating osteoporosis by promoting low‐grade chronic inflammation and regulating MSC lineage commitment.^57^ ω‐3 LCPUFAs inhibit osteoclastogenesis, decrease PGE2 content, and thus increasing BMD to benefit osteoporosis prevention and alleviation.^51^, ^58^ In addition, fatty acids such as palmitate could enhance energy generation for osteoblast differentiation, thus accelerating bone formation.^52^ Moreover, since LCPUFAs are highly prone to reactive oxygen species (ROS)‐induced oxidative damage, adoption of antioxidant CoQ as adjuvant could eliminate the disadvantages of LCPUFAs during osteoporosis therapeutics.^59^, ^60^

Estrogen deficiency‐induced postmenopausal osteoporosis is the most common type of osteoporosis. Along with decrease in estrogen levels, reduction in OPG delays osteoblast maturation and attenuates bone formation^61^; also, drop in OPG/RANKL ratio enhances osteoclast differentiation and promotes bone resorption and eventually results in bone loss.^47^ Moreover, endogenous fatty acids could serve as energy sources of skeletal and bone marrow cells to contribute to postmenopausal women bone health,^62^ while exogenous supply of fatty acids might favour or harm postmenopausal women bone condition. As illustrated by animal models, supplementation of fatty acids such as and SCFAs^63^ and ω‐3 LCPUFAs^56^ substantially reduces bone loss and restores bone mass and thus ameliorates postmenopausal bone loss in ovariectomized mice, and the protective roles of SCFAs on bone loss were mainly attributed to the suppression of osteoclast differentiation and function.^64^ According to data derived from human subjects, although earlier investigations indicate that ω‐3 LCPUFAs intake plays positive roles in enhancing bone mass and limiting postmenopausal osteoporosis risks,^65^ effects of PUFAs on bone are shown to be contradictory in general. For example, there is one study demonstrated that PUFA supplementation significantly enhanced lumbar spine and femoral neck BMD in a population of 65 postmenopausal women; however, another trial reported no significant therapeutic effects in 42 postmenopausal women receiving similar PUFA supplements.^56^ Taken together, understanding the functions and mechanisms of fatty acids in osteoporosis might help to develop novel preventive or therapeutic strategies to benefit bone health maintenance in osteoporotic patients.^60^, ^66^

### 2.3 Bone fracture

The high risk of bone fractures may result from osteoporosis with low BMD, or more specifically, deterioration of bone structure and loss of bone mass.^67^, ^68^, ^69^ Studies in mice models have suggested that endogenously produced ω‐3 LCPUFAs could facilitate fracture healing process, and supplementation of ω‐3 LCPUFAs exert positive effects on fracture healing.^21^ Consistently, investigations in human subjects by Sadeghi *et al* 68 and Harris *et al* 67 have indicated that increased intake of total PUFAs is positively correlated with higher BMD and reduced bone fracture risk in populations including elder men. However, epidemiological investigation by Virtanen *et al* demonstrates that low total PUFA, ω‐6 PUFA or LA intakes might promote the risk of hip fractures in women.^70^ Apart from heterogeneity in study design, sample inclusion and data process among different studies, diversity in fatty acid types might be an important factor contributing to the conflicting involvement of fatty acids in bone fractures. Correspondingly, specific mechanisms of fatty acid modulation on bone fractures vary a lot. For example, ω‐6 LCPUFAs such as arachidonic acid (AA) could stimulate PGE2 production to regulate bone metabolism and fracture healing, while ω‐3 PUFAs increase BMD by increasing calcium resorption and bone collagen synthesis, decreasing urinary calcium excretion, and thus inhibiting bone resorption.^67^, ^68^ Overall, fatty acids of different types might exert differential effects on bone fractures pathophysiology, and much more work needs to be done on exploiting them for bone fractures prevention and therapeutics.

### 2.4 Rheumatoid arthritis

Rheumatoid arthritis, with manifestations of arthralgia, redness and swelling, and limited range of motion,^71^ is a chronic and autoimmune inflammatory disease affecting 0.5%−1% of the world population.^72^, ^73^, ^74^ If left untreated or ineffectively treated, rheumatoid arthritis typically leads to primary joints destruction caused by erosion of cartilage and bone, as well as subsequent systemic complications and even death.^72^, ^73^, ^74^, ^75^ Several studies have investigated the individual and combinational protective effects of LCPUFAs in rheumatoid arthritis. For example, ω‐3 LCPUFAs could lower the risk of cardiovascular disease in rheumatoid arthritis patients,^23^ and combinational utilization of ω‐3 LCPUFAs with low‐dose vitamin E could substantially reduce the side effects of disease‐modifying anti‐rheumatic drugs (DMARDs).^75^ The attenuation effects of ω‐3 LCPUFAs on rheumatoid arthritis‐induced bone and cartilage destruction are mainly mediated by reduced synthesis of cartilage‐degrading enzymes as well as the inflammatory response cytokines. ω‐3 LCPUFAs, especially EPA and DHA,^23^, ^76^ could alleviate morning stiffness and decrease number of swollen and tender joints in patients with rheumatoid arthritis and thus show anti‐inflammatory and restorative effects against rheumatoid arthritis. Importantly, since LCPUFAs AA could drive the synthesis of pro‐inflammatory cytokines, restriction of AA enhances ω‐3 LCPUFAs‐mediated anti‐inflammatory responses by decreasing the production of metalloproteinases and pro‐inflammatory cytokines as well as the migration of leucocytes in vivo, and thus strengthens the action of ω‐3 LCPUFAs in combating rheumatoid arthritis.^75^, ^76^ Another kind of LCPUFAs, ω‐6 LCPUFAs are eventually metabolized into AA and inflammatory eicosanoids and function as pro‐inflammatory agents,^75^, ^77^ ω‐3 LCPUFAs could reduce the synthesis of ω‐6 LCPUFAs by competing with the rate‐limiting delta‐6 desaturation enzyme and thus exert a therapeutic effect on rheumatoid arthritis.^75^, ^78^, ^79^ Moreover, SCFAs also play crucial roles in bone metabolism and immune responses in pathological bone loss and thus regulate systemic bone mass and protect from rheumatoid arthritis.^64^ Investigations are needed to further elucidate mechanisms underlying the pharmacological roles and therapeutic potentials of multiple types of fatty acids in arthritis such as temporomandibular joint arthritis.^66^

### 2.5 Tumour‐associated bone destruction

Multiple myeloma is a destructive cancer that mainly occurs in bone marrow.^80^ Studies have shown that fatty acids of different types play either pro‐death or pro‐survival roles in multiple myeloma. For example, PA could activate apoptosis in multiple myeloma cells and thereby serves as a potentially direct anti‐myeloma strategy.^81^ EPA and DHA could also initiate apoptosis and promote drug sensitivity in multiple myeloma cells, with a mechanism involving NF‐κB inhibition concomitant with activation of mitochondrial defects leading to caspase‐3 activation and apoptosis.^82^ In addition, EPA and DHA modulate p53/miR‐34a/Bcl‐2 axis to enhance dexamethasone (Dex)‐sensitivity in multiple myeloma cells where they trigger p53 expression and subsequent increase of miR‐34a levels in U266 cells, and finally activate Bcl‐2 to induce apoptosis of multiple myeloma cells.^83^, ^84^, ^85^ By contrast, SFAs and ω‐6 LCPUFAs represent energy sources for multiple myeloma cells, and ratio of ω‐3/ω‐6 fatty acid intake is critical for the maintenance of multiple myeloma cell survival.^86^, ^87^

Bone metastasis is a pernicious complication^88^ occurring in virtually 60% of patients with osteolytic breast or osteogenic prostate cancers and at a smaller rate in patients with other cancer types.^89^, ^90^ Patients with bone metastasis suffer from severe pain, bone fracture and osteolytic lesions, which symptoms are primarily attributed to aberrant bone resorption mediated by osteoclasts.^91^, ^92^ In osteolytic metastasis mice model originating from MDA‐MB‐231 human breast cancer, researchers found that supplementation with DHA and EPA‐enriched fish oil prevented breast cancer metastasis‐induced bone osteolysis,^93^ suggesting potential therapeutic effects of fatty acids for osteolytic bone metastasis. In mechanism, both DHA and EPA reduce the mRNA and protein levels of CD44 in breast cancer cells to inhibit cancer invasion; moreover, compared to EPA, DHA has profound anti‐inflammatory effects via inhibiting TNF‐α secretion and NF‐κB activation in macrophages and thus exhibits stronger suppression of osteoclast activity to attenuate the related osteolysis.^94^ However, in osteogenic metastasis derived from prostate cancer, fatty acids such as AA could facilitate metastatic cancer cell implantation and propagation via preparation of bone microenvironment “soil” for cancer cells by activating bone marrow adipocyte formation,^95^ demonstrating promotional roles of fatty acids in favour of osteogenic bone metastasis. This might be explained by the fact that fatty acids synthesized by bone marrow adipocytes could serve as energy source for certain types of tumour cells engaged in metastasis. During bone metastasis of prostate cancer, free fatty acid influx into cells induces the expression of lipid transport mediator fatty acid‐binding protein (FABP4), and expression of FABP4 between tumour cells and adipocytes could mediate adipocyte‐induced metabolic switch in prostate microenvironment and thus promotes osteogenic prostate cancer metastasis.^96^ Such roles of fatty acids in facilitating bone metastasis have also been confirmed in melanoma cancer,^89^ where bone marrow adipocytes play a pivotal role in bone metastasis by releasing free fatty acids to meet the energy demands of metastatic cancer cells for survival and growth. Therefore, fatty acids of different types behave significantly differently in cancer bone metastasis, and osteolytic or osteogenic or mixed bone lesion conditions derived from specific cancer types should be definitely taken into account when employing fatty acids for cancer bone metastasis therapeutics.

### 2.6 Other bone disorders

Fatty acids are also involved in non‐typical skeletal diseases such as osteomyelitis, a bone inflammatory process initiated by infection of pyogenic organisms 97 that predominantly occurs in long bones of children, and in hips, feet, jaws and spine of adults.^98^, ^99^, ^100^ This disease is characterized by severe damage to bone tissue and bone marrow, and probably accompanied by high morbidity and mortality.^100^ Accumulating evidence has shown that ω‐3 LCPUFAs could effectively combat microbial pathogenesis in osteomyelitis.^101^, ^102^, ^103^ Furthermore, combination of vancomycin and ω‐3 LCPUFAs has been suggested to be a reliable therapeutic strategy against *S aureus*‐induced osteomyelitis, with a mechanism involving inflammation alleviation by reducing TNF‐α and interleukin 6 (IL‐6) levels as well as antioxidant activity by decreasing SOD activity.^97^

**Figure 2:**
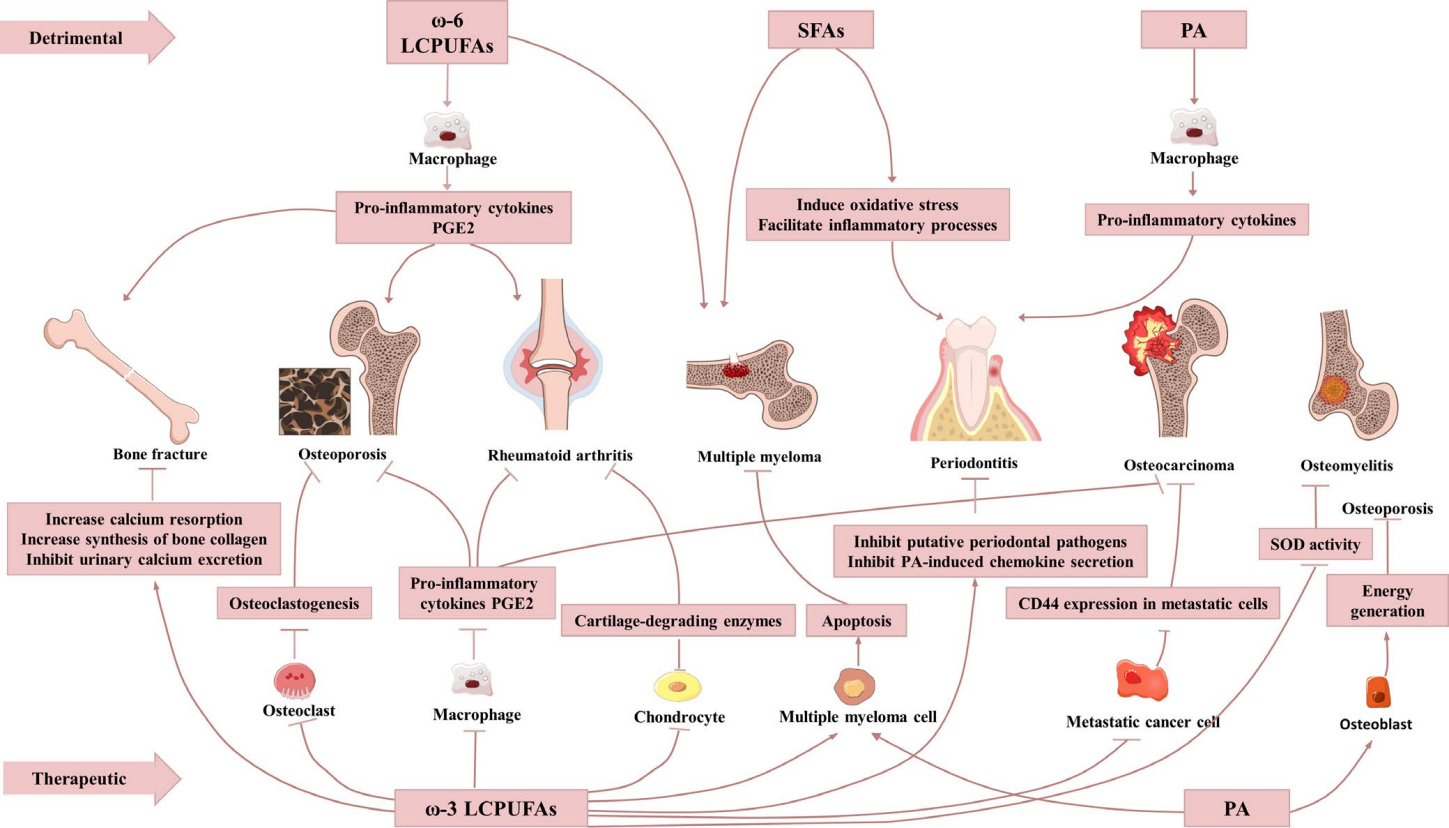
Implications of fatty acids in bone diseases. Fatty acids exert dual effects on bone either by alleviating or initiating bone diseases. ω‐6 LCPUFAs, SFAs and PA predominantly promote pathological bone remodelling by facilitating pro‐inflammatory processes and lead to osteoporosis, rheumatoid arthritis, periodontitis, *etc* Other fatty acids like ω‐3 LCPUFAs have therapeutic value in blocking bone disorders. Their targets include bone tissue components such as osteoblasts, osteoclasts, macrophages, chondrocytes and aberrant multiple myeloma cells, metastatic cancer cells, suppressing skeletal inflammation, carcinoma and bone fracture through complicated mechanisms. LCPUFAs, long‐chain polyunsaturated fatty acids; SFAs, saturated fatty acids; PA, palmitic acid

Taken together, according to currently available pre‐clinical experiments (Table 2) and clinical studies (Table 3), various factors contribute to implications of different fatty acids types in multiple bone disorders. With most associations between fatty acids and bone disorders remain obscure (Figure 2), much more work needs to be done by collaboration of biological and clinical researchers to maximize the therapeutic potentials and minimize the side effects of fatty acids against bone diseases.

**Table 2:** Animal experimental studies evaluating effects of fatty acids in bone disorders

Class	Disease	Animal	Treatment	Study period	Bone‐related outcome	Conclusion	Year	Reference
ω‐3 LCPUFAs	Periodontitis	Male Wistar rats	Control group (C) Group 1：ω‐3 PUFAs (C + O) Group 2: pulp exposure‐induced apical periodontitis (AP) Group 3: pulp exposure‐induced AP + ω‐3 PUFAs (AP + O)	45d	Areas of bone resorption/inflammatory intensity ：AP group ＞ AP + O, C + O and C groups	ω‐3 LCPUFAs decrease inflammatory cell infiltration and AP bone resorption	2018	35
	Osteoporosis	Male piglets	Diet: suckling/standard formula/formula containing LCPUFAs Drug: placebo/ dexamethasone (DEX)	15d	DEX group: BMC of whole body, femur, and lumbar spine ↓ Suckled group: highest BMC of femur and whole body LCPUFA group: PGE2↑	ω‐3 LCPUFAs give rise to BMC of femur and whole body	2002	277
	Osteoporosis	c57Bl/6 mice	High‐fat diet/normal control	24w	Trabecula number and surface↑ Trabecular separation↓	HFD‐induced obesity promotes bone formation	2010	278
	Osteoporosis	Fat‐1 mice	Group 1: Fat‐1 mice sham Group 2: Fat‐1 mice ovariectomized (OVX) Group 3: WT sham Group 4: OVX	5mo	Bone marrow adiposity↓ Bone parameters↑in the distal femoral metaphysis	ω‐3 LCPUFAs improve osteoblastogenesis to treat osteoporosis	2013	279
	Osteoporosis	Fat‐1 mice	Ovariectomized (Ovx) and sham operated AIN‐93M diet containing 10% corn oil	24w	Osteotropic factors↓ BMD↑	ω‐3 LCPUFAs effectively prevent post‐menopausal osteoporosis	2009	280
	Aging‐related bone loss	Gonad‐intact middle‐aged male rats	Group 1: ω‐6 + ω‐3 diet (control) Group 2: ω‐6 diet (almost devoid of ω‐3 LCPUFA) Group 3: ω‐3 diet (rich in ω‐3 LCPUFA)	20w	Group 1:BMD↓ Group 2: bone PGE2 production↑ Group 3: bone‐specific alkaline phosphatase activity↑ + highest bone mineral and BMD	ω‐3 LCPUFAs protect gonad‐intact middle‐aged male rats from bone loss	2005	281
	Aging‐related bone loss	Male Wistar rats	Diet: virgin olive oil/ sunflower oil/ (ω‐6 LCPUFAs)/ fish oil (ω‐3 LCPUFAs)	24mo	Bone loss：sunflower oil (+++) fish oil (++) virgin olive oil (+)	Dietary ω‐3 LCPUFAs prevent aging‐associated bone loss ω‐6 LCPUFAs prevent aging‐related alveolar bone loss	2013	282
	Aging‐related bone loss	Female Polycystic kidney disease (PKD) rats	Group 1:casein + corn oil (Casein + CO) Group 2: casein + soybean oil (Casein + SO) Group 3: soy protein isolate + soybean oil (SPI + SO) Group 4: soy protein isolate + 1:1 soybean oil:salmon oil blend (SPI + SB)	12w	Femur length: SPI + SO ＜ Casein + CO	ω‐3 LCPUFAs influence bone longitudinal growth and mineral balance	2015	283
	Aging‐related bone loss	Male Wistar rats	Group 1: fish oil Group 2: fish oil + coenzyme Q10 (CoQ10)	24mo	Aged rats bone mineral density: group 1＜group 2	CoQ10 avoids aging‐related bone loss	2017	59
	Osteoarthritis	Guinea pigs	High ω‐3 diet/typical western diet	20w	ω‐3 diet group: OA average histological scores↓; cartilage parameters modified	ω‐3 LCPUFAs decrease OA in prone strain and increase no marker of pathology in either strain	2011	284
	Bone fracture	Fat‐1 ± C57BL/6 mice	Diets containing 10% corn oil	12w	Fat‐1 + C57BL/6 mice exhibited acceleration in endochondral ossification, callus formationand remodeling process compared to fat‐1 ‐C57BL/6 mice group	ω‐3 PUFAs positively affect fracture healing	2017	21
	Bone growth	Female white rabbits	Diet: soy bean oil (SBO control)/sesame oil (SO)/fish oil (FO)/algae oil	100d	Bone marrow fatty acids ↑ FO diet：highest ω‐3 LCPUFAs SBO diet：highest ω‐6 LCPUFAs	ω‐6/ω‐3 LCPUFAs ratios are involved in bone resorption decrease and bone mass improvement during growth	2014	285
	Bone growth	Post‐partum female Wistar rats	Diet: flaxseed flour + semi‐purified diet	51d	Bone maximum force/breaking strength/ rigidity/ femoral head radiodensity ↑	ω‐3 LCPUFAs enhance bone density and bone strength	2017	286
	Cancer bone metastasis	Immune‐compromised (nu/nu) mice	Lab chow diet/fish oil + intracardiac injection of the MDA‐MB‐231 cells	6w	Fish oil diet group:osteolytic lesions ↓; migration of breast cancer cells↓; CD44 expression ↓	ω‐3 LCPUFAs prevent breast cancer bone metastasis	2011	93
	Cancer bone metastasis	Female BALB/c mice	ω‐3/ω‐6 LCPUFAs diet + orthotopic implantation of 4T1 mammary tumor cells	147d	Bone metastases frequencies: ω‐3 group＜ω‐6 group	Dietary ω‐3 LCPUFAs reduce tumor metastasis to bone	2018	287
ω‐6 LCPUFAs	Periodontitis	C57BL/6 mice	Group 1: palmitic acid (PA)‐enriched high‐fat diet Group 2: oleic acid (OA)‐enriched high‐fat diet Group 3: normal caloric diet	16w	Weight↑（group 1 and group 2） alveolar bone loss and TNF‐α levels: group 1 (+++) bone remodeling markers: group 3 (+++) group 2 (++) group 1 (+)	PA aggravates alveolar bone loss and osteoclast inflammation	2016	38
	Aging‐related bone loss	Male Wistar rats	Diet: virgin olive oil/sunflower oil/(ω‐6 LCPUFAs)/fish oil (ω‐3 LCPUFAs)	24mo	Bone loss：sunflower oil (+++) fish oil (++) virgin olive oil (+)	ω‐3 PUFAs prevent aging‐related bone loss ω‐6 LCPUFAs are associated with aging‐related alveolar bone loss	2013	282
	Aging‐related bone loss	Male Wistar rats	Diet: virgin olive oil (V group)/ sunflower oil (S group)	24mo	Bone Mineral Density/Bone Mineral Content/ Bone Areal Size：V group＞S group	MUFAs prevent aging‐related BMD decrease	2017	288
	Osteoarthritis	Female mice	Group 1: C2/C3/C4 supplementation Group 2: fibre‐rich diet Group 3: bacterial transfer	8w	Group 1: bone volume per tissue volume↑， trabecular separation↓ Group 2: systemic bone mass↑, trabecular separation↓ Group 3: osteoclast numbers↓， systemic bone mass↓	SCFAs regulate bone metabolism to optimize arthritis severity	2018	64
	Osteoarthritis	8‐week‐old female mice	Group 1: C2/C3/C4 supplementation Group 2: fibre‐rich diet Group 3: Prevotella transfer into WT mice	8w	Group 1: bone volume per tissue volume↑， trabecular separation↓ Group 2: systemic bone mass↑, trabecular separation↓ Group 3: osteoclast numbers↓， systemic bone mass↓	SCFAs regulate bone metabolism and immune responses to alleviate arthritis	2018	64
	Partum‐related bone loss	Wistar rats	Control goup: placebo Test group: flaxseed flour	51d	Test group: arachidonic acid (ARA)↓ alpha‐linolenic acid (ALA) eicosapentaenoic (EPA)↑ femoral head radiodensity↑	ALA together with calcium increase bone density in post‐partum period	2017	286
SFAs	Periodontitis	Male C57BL/6 mice	Diet：regular chow/ high‐fat diet	16w	LPS‐induce alveolar bone loss↑ LPS‐induce osteoclastogenesis ↑	SFAs are potentially involved in MetS‐related periodontitis	2015	36
	Osteoarthritis	Male rats	H group:20% beef tallow HLA group: 20% lauric acid HAS group: 20% stearic acid	16w	Articular cartilage degeneration Bone architecture changes Average osteocyte lacunae↓	SFAs prevent OA development	2017	260
MUFAs	Periodontitis	C57BL/6 mice	Group 1: PA‐enriched high‐fat diet Group 2: OA‐enriched high‐fat diet Group 3: normal caloric diet	16w	Weight↑（group 1 and group 2） alveolar bone loss and TNF‐α levels: group 1 (+++) bone remodeling markers: group 3 (+++) group 2 (++) group 1 (+)	OA can aggravate the alveolar bone loss and inflammation of osteoclasts	2016	38

**Table 3:** Epidemiological and clinical studies evaluating effects of fatty acids in bone disorders

Class	Disease	Intervention	Study Period	Enrolment	Bone‐related outcome	Conclusion	Year	Reference
ω‐3 LCPUFAs	Periodontitis	Control group: placebo Test group: DHA	3mo	55	IL‐1β↓ mean pocket depth↓ gingival index↓	DHA greatly contributes to moderate periodontitis and gingival inflammation	2014	45
	Periodontitis	Group 1: EPA 500 mg Group 2: borage oil 500 mg Group 3: EPA 500 mg and borage oil 5oo mg	12w	30	Periodontal probing depth and gingival inflammation group 1 (+) group 2 (+++) group 3 (++)	Borage oil have better influences on periodontal inflammation than EPA	2003	289
	Periodontitis	Control group: decalcified freeze dried bone allograft (DFDBA) + placebo Test group: DFDBA + omega‐3 polyunsaturated fatty acids combined with low‐dose aspirin o	6mo	40	Probing pocket depth↓(T) IL‐1b and IL‐10↓(T)	ω‐3 LCPUFAs combined with low‐dose aspirin decrease gingival inflammation, pocket depth and attachment level gain	2011	290
	Rheumatoid arthritis	Control group: placebo Test group: daily liquid nutrient supplementation	4mo	66	EPA, DHA and docosapentaenoic acid↑ arachidonic acid↓	EPA and GLA do not benefit RA patients at test doses	2004	291
	Rheumatoid arthritis	Control group: diet group regarding the fatty acid intake Test group: Mediterranean diet	52w	13	Ratio of ω‐6 to ω‐3 fatty acids↓ intake of ω‐3 fatty acids↑	Revealed by dietary assessments and through fatty acids in s‐phospholipids, the fatty acid profile is different in the Cretan Mediterranean diet	2005	292
	Rheumatoid arthritis	2 mL/kg fish oil emulsion intra‐ venously	5mo	34	Short‐term efficacy↑ rapid onset excellent tolerability	ω‐3 PUFAs are safe and effective for RA	2006	293
	Rheumatoid arthritis	Drug: cod liver oi Drug: placebo	9mo	97	Daily NSAID requirement↓	ω‐3 LCPUFAs decrease NSAID‐ sparing agents	2008	294
	Rheumatoid arthritis	Control group: placebo Test group: Step 1:0.2g of fish oil emulsion/kg intravenously Step 2:0.05 g of fish oil/kg orally	14d	23	Swollen joint count↓ Tender joint count↓	ω‐3 LCPUFAs improve symptoms of RA and extend the beneficial effects of infusion therapy	2010	295
	Rheumatoid arthritis	Drug: ω‐3 LCPUFAs Drug: Placebo	12w	60	Clinical benefit concomitant analgesic medication↓ no weight change	ω‐3 LCPUFAs decrease use of concomitant analgesic without weight changes	2015	75
	Rheumatoid arthritis	High‐dose/Low‐dose fish oil + disease‐modifying anti‐rheumatic drug(DMARD)	12mo	140	Failure of DMARD therapy ↓	ω‐3 LCPUFAs increase RA remission and decrease DMARD therapy failure	2015	296
	Rheumatoid arthritis	RA‐free participants at increased risk for RA	10y	136	Percent of ω‐3 LCPUFAs in red blood cells↑→ rheumatoid factor (RF) positivity in shared epitope (SE)‐positive participants↓	ω‐3 LCPUFAs exert pronounced effects on RA‐related autoimmunity	2017	297
				2166	ω‐3 LCPUFAs supplement use↑→RF positivity in SE‐positive participants↓			
ω‐6 LCPUFAs	Periodontitis	Group 1: EPA 500 mg Group 2: borage oil 500 mg Group 3: EPA 500 mg and borage oil 5oo mg	12w	30	Periodontal probing depth and gingival inflammation group 1 (+) group 2 (+++) group 3 (++)	Borage oil has better effects on periodontal inflammation than EPA	2003	289
SCFAs	Periodontitis	Periodontal treatment,	6mo	21	Levels of formic acid↑ Levels of lactic acid, propionic acid, butyric acid and isovaleric acid↓	Formic acid in gingival crevicular fluid is inversely associated with periodontitis severity Butyric and isovelaric acids can indicate development and progression of periodontitis	2012	39

## 3 SIGNALLING PATHYWAYS INVOLVED IN FATTY ACIDS‐MODULATED BONE METABOLISM

Fatty acids and their metabolites could modulate bone metabolism via mechanisms such as inflammation,^104^ apoptosis,^105^ autophagy 106 and oxidative stress.^104^ Normally, fatty acids bind to specific cellular membrane‐bound or nucleus‐located targets, induce subsequent transduction of transmembrane/nucleus‐specific signals, further result in modulation of target gene transcription and protein synthesis and finally contribute to the regulation of cell growth, behaviour and function. Given that a multitude of factors are involved in these processes, understanding the underlying mechanisms will substantially facilitate the nutritional and therapeutic applications of fatty acids in bone homeostasis and disorders.

### 3.1 Receptors involved in fatty acids‐modulated bone metabolism

Cellular membrane‐bound and nuclear receptors, such as G protein‐coupled receptors (GPRs), peroxisome proliferator‐activated receptors (PPARs), TLRs and receptors for metabolites such as chemokine‐like receptor (ChemR), play essential roles in mediating the effects of fatty acids on bone metabolism.

GPRs are a superfamily of more than 1000 distinct membrane receptors; several GRPs among these have been reported to be modulated by fatty acids. GPR18, GPR41, GPR43 and GPR109A are receptors for SCFAs (C2‐C5) found in both osteoclasts and osteoblasts, where GPR41 could regulate leptin production, while GPR43 is the main receptor in mediating effects of SCFAs on osteoclasts.^30^, ^107^, ^108^ GPR40, which is expressed on osteoclasts and could be activated by medium/long‐chain fatty acids with a chain length of C8‐C22,^109^, ^110^, ^111^ positively affects bone metabolism by downregulating osteoclastogenesis, combating bone loss and protecting cartilage.^112^, ^113^, ^114^ GPR84, whose expression in macrophages and adipocytes could be enhanced under inflammatory conditions, is a receptor for MCFAs (C9‐C14).^115^, ^116^, ^117^ GPR120, which is expressed on osteoblasts and osteoclasts and could be stimulated by long‐chain saturated (C14‐C18) and long‐chain unsaturated fatty acids (C16‐C22),^109^, ^110^, ^111^ has been shown to mediate the anti‐inflammatory effects of DHA in macrophages.^118^ And GRP120 could enhance ω‐3 LCPUFAs‐induced osteoblastic bone formation by inducing β‐catenin activation and reduce osteoclastic bone resorption by suppressing NF‐κB signalling,^14^ and GPR120 could also modulate the bi‐potential differentiation of BMMSC in a dose‐dependent manner.^119^ In addition to the acknowledged roles of GPR40 and GPR120 in preventing bone disorders such as osteoporosis and osteoarthritis,^120^ GPR2 family member parathyroid hormone type 1 receptor (PTH1R) also plays a role in bone metabolism. PTH1R could mediate ω‐3 LCPUFAs‐induced activation of extracellular signal‐regulated kinases (ERK) to enhance osteoblasts proliferation and differentiation^121^, ^122^; moreover, EPA and DHA could act as agonists of PTH1R to attenuate osteoblast apoptosis and promote bone formation.^123^

PPARs, with known ligands including LCPUFAs and metabolites such as PGE2, are nuclear receptors that regulate lipid metabolism by acting as transcription factors in BMMSCs, osteoblasts and osteoclasts.^124^, ^125^, ^126^, ^127^, ^128^ When BMMSCs are exposed to a mixture of palmitic, oleic and linoleic acids, upregulation of PPARs and reduction of Runx2 facilitate differentiation of towards adipocyte‐like cells.^129^ Influences of PPARs on osteoblasts and osteoclasts depend on specific receptor isoform. Specifically, PPARα/β promotes bone resorption,^130^ whereas PPARγ is known inhibitors of osteoclastogenesis.^131^ Roles of PPARγ in osteoblasts are still disputed; it has been shown that conditional deletion of PPARγ in osteoblasts enhances bone mass and increased bone formation by activating mTOR signalling,^132^ while studies from other groups reported conflicting results.^133^, ^134^, ^135^ Moreover, recent findings have indicated that treatment of multiple myeloma cells with PPARs resulted in apoptotic effects,^136^ suggesting PPARs might serve as promising therapeutic targets for bone diseases.

TLRs mainly mediate the inflammatory action of fatty acids in bone cells. PA particularly activates TLR2 and induces IL‐1β expression and secretion to promote inflammatory response.^137^, ^138^, ^139^ Binding of SFAs to TLR4 on osteoclasts induces chronic inflammation 140, 141, 142 by enhancing the expression of macrophage inflammatory protein‐1a, which leads to hyperactivation of NF‐κB and subsequent enhancement of osteoclastic activities 143 as well as further decrease in bone size, BMC and BMD.^144^ Moreover, studies have shown that DHA treatment could block the pro‐inflammatory effects of lauric acid‐induced TLR2/4 activation in Raw264.7 cells,^145^ suggesting TLRs might be involved in the crosstalk among multiple downstream signalling pathways of different fatty acids types.

ChemR23 can act as chemerin receptor 146, 147 as well as RvE1 receptor in bone tissue cells such as monocytes.^148^ Binding of RvE1with ChemR23 could prevent inflammation by inhibiting NF‐κB activation,^149^ enhancing bone formation^150^ and reducing bone loss via RANKL/OPG ratio modulation,^151^, ^152^ while the detailed mechanisms involved in the downstream of Chem23 have yet to be fully elucidated.

### 3.2 RANK/RANKL/OPG signalling in fatty acids‐modulated bone metabolism

**Figure 3:**
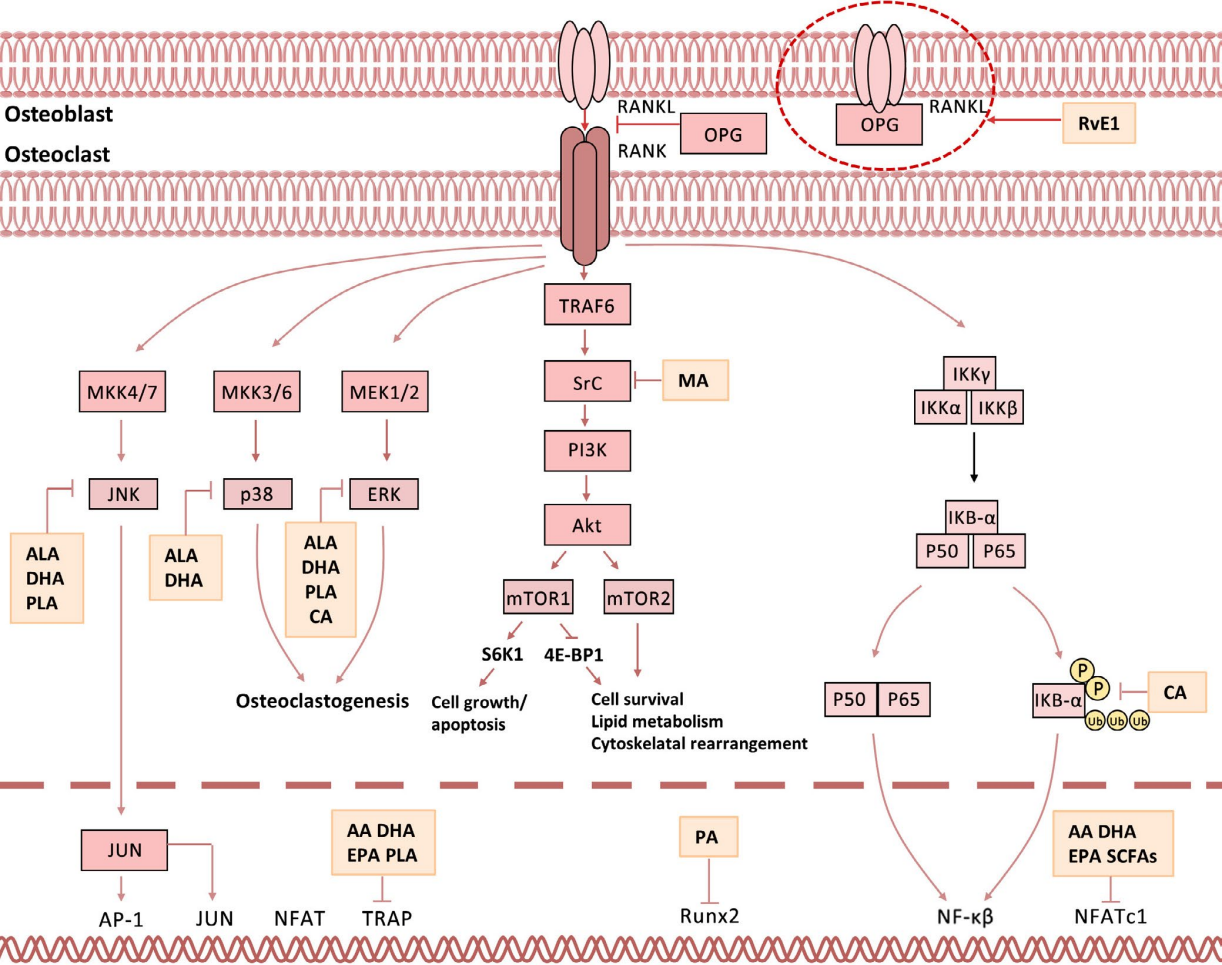
RANK/RANKL/OPG pathway in fatty acids‐modulated bone metabolism. The well‐documented RANKL signalling pathway exerts essential role in osteoclastogenesis. RANKL binds to RANK on the surface of osteoclast precursor cells and activates three distinct downstream signalling pathways. The MAPK pathways characterized by downstream factors ERK, p38 and JNK play pivotal role in cell death and survival. The NF‐κB signalling pathway is activated following IκBα phosphorylation and degradation. The p50 and p65 subunits of NF‐κB are released and translocated into the nucleus to activate the transcription of target genes. The PI3K/mTOR pathway is also activated upon binding of RANKL to RANK, which triggers the activation of PDK1s and Akt leading to the inhibition of the TSC complex and subsequent mTORC1 formation. The mTORC1 phosphorylates S6K1 as well as 4E‐BP1, which further regulate protein synthesis, cell proliferation, angiogenesis and autophagy. However, mTORC2 acts as an essential modulator of actin cytoskeleton, cell survival and lipid metabolism. RANKL, receptor activator of nuclear kappa‐β ligand; TGF‐β, transforming growth factor β; JNK, c‐jun NH2‐terminal kinase; Akt, protein kinase B; S6Ks, S6 kinases; 4E‐BP1, 4E‐binding protein 1

To our knowledge, various signalling pathways including RANKL,^153^ NF‐κB,^154^ mitogen‐activated protein kinase (MAPK),^155^ Wnt,^156^ Notch,^157^ Hedgehog,^158^ transforming growth factor‐β (TGF‐β),^155^ mTOR 159 and bone morphogenetic protein (BMP)^155^ are involved in bone metabolism. Among these, RANK/RANKL/OPG signalling is most frequently implicated in bone remodelling via modulation by a wide variety of fatty acids^160^, ^161^ (Figure 3). Upon activation, RANK/RANKL/OPG signalling substantially inhibits osteoclastogenesis but enhances osteogenesis via downstream signalling cascades such as MAPK, NF‐κB and phosphatidylinositol 3‐kinase (PI3K)/mTOR.^160^ Specifically, MAPK signalling^162^, ^163^, ^164^, ^165^ could be activated by ω‐7 LCMUFAs,^166^ PA and MCFAs^167^ and activation of MAPK signalling normally leads to enhanced proliferation of both osteoblasts and chondrocytes.^155^, ^163^, ^165^ By contrast, ALA,^168^ ω‐7 LCMUFAs^166^ and MCFAs^167^ could inhibit NF‐κB cascade, and repression of NF‐κB cascade attenuates osteoclastogenesis by enhancing both cell death and differentiation.^154^, ^165^, ^169^, ^170^ Moreover, PI3K/mTOR pathway could be downregulated by EPA or LXA_4_ but upregulated by PA^159^ and thus involved in BMMSC differentiation, osteoblast function and osteocyte formation during bone metabolism.^171^, ^172^, ^173^, ^174^, ^175^, ^176^, ^177^

## 4 MODULATION OF FATTY ACIDS ON SPECIFIC BONE CELL TYPES

### 4.1 Fatty acids and osteoblasts

Osteoblasts are mononuclear cells predominantly involved in bone formation 4, 5. A growing body of evidence supports the promotional or inhibitory action of fatty acids on osteoblasts. In general, fatty acids such as PA suppress osteoblast function, whereas EPA, DHA and RvE1 predominantly promote osteoblastic function. Exploring the modulation effects of fatty acids on osteoblasts might provide new insights into therapeutic intervention targeting skeletal disorders associated with dysregulated bone formation.

#### 4.1.1 Fatty acids as negative regulators of osteoblasts

Palmitate, a kind of LCSFAs, impedes osteoblast differentiation and induces cell death *via* lipotoxicity.^105^ Palmitate could induce autophagy in osteoblasts dependent on Beclin and PI3K,^178^ and autophagy serves as a protection mechanism in preserving osteoblasts from lipotoxicity.^179^ Palmitate also promotes apoptosis of osteoblasts through both extrinsic and intrinsic pathways, and PA‐induced high expression of cytosolic cytochrome C could be disrupted by inhibition of c‐Jun N‐terminal kinase (JNK).^105^ In foetal rat calvarial cell cultures, palmitate affects neither proliferation nor apoptosis of calvarial cells but represses BMP‐7‐induced osteoblastic differentiation by reducing the activity of transcription factor SMAD, and thus further abrogating expression of osteogenic markers Runx2, osteocalcin, alkaline phosphatase and bone sialoprotein.^180^ Interestingly, enhancing fatty acid oxidation could block all lipotoxic effects of palmitate suggested above, indicating that fatty acid oxidation might relieve the negative effects of palmitate on osteoblasts.^105^

#### 4.1.2 Fatty acids as positive regulators of osteoblasts

##### LCPUFAs and SCFAs

ω‐3 LCPUFAs such as EPA and DHA could stimulate osteoblasts survival by activating pro‐survival Akt signal and suppressing glucocorticoid‐induced pro‐death pathway.^123^ They also promote osteoblastogenesis and prevent bone resorption by altering membrane function, regulating calcium balance and enhancing osteoblast activity.^57^ Involvement of EPA and DHA in preosteoblasts differentiation and maturation is largely associated with their anti‐inflammatory effects, which function by reducing the synthesis of inflammatory ARA‐derived PGE2,^181^ modulating PPAR‐γ signalling and thus lower levels of inflammatory cytokines such as IL‐1, IL‐6 and TNF‐α,^182^ and suppressing AA‐derived synthesis of eicosanoids^183^ as well as activity of cyclooxygenase and 5‐lipoxygenase.^184^ Therefore, as illustrated above, intake of EPA and DHA might have potent therapeutic implications in inflammatory bone disorders such as osteoporosis.^24^

ω‐6 LCPUFAs are activators of PPARγ, and lower dietary ratio of ω‐6/ω‐3 LCPUFAs blocks PPAR‐γ activation and thus enhancing osteoblastogenesis.^56^ Besides, SCFAs such as butyrate promote osteoblast formation and differentiation by enhancing production of bone sialoprotein and osteopontin; moreover, it stimulates osteoblasts to secret OPG and thus facilitating the blocking of osteoclast differentiation.^185^

##### Fatty acids derivatives

RvE1 is an EPA metabolite that is closely associated with inflammation‐induced bone disorders. In IL‐6‐stimulated osteoblasts, supplement of RvE1 leads to significant disruption of PI3K‐Akt pathway, which interacts with NF‐κB, MAPK and p53 signalling to modulate protein synthesis, cell differentiation and apoptosis. In inflammatory bone disorders, changes in production of pro‐inflammatory cytokines such as TNF‐α, IL‐6, IL‐1 and Gas6 186 modulate RANKL/OPG ratio and downstream events^151^, ^152^ and thus enhance osteoclasts‐mediated pathological inflammation‐induced bone resorption.

As a metabolite of AA, PGE2 exerts its effects on BMMSCs, osteoblasts and osteoclasts in dose‐dependent manner. In BMMSCs, PGE2 binds with EP4 receptor, which activates sphingosine kinase and inhibits caspases activities and thus prevents apoptosis of BMMSCs.^187^ In osteoblasts, intermittent administration,^188^ short‐term exposure to high doses or prolonged treatment with lower doses of PGE2^189^ could enhance proliferation and activity of osteoblasts and lead to remarkably enhanced bone formation; such influences of PGE2 on osteoblasts are mediated by EP2/EP4‐MAPK signalling pathways.^190^ In addition, effects of PGE2 on osteoblasts are accompanied by osteoclast stimulation, which might reverse the overall influence of PGE2 on bone system.^191^

### 4.2 Fatty acids and osteoclasts

Osteoclasts are multinucleated giant cells with bone resorptive activity. Two essential factors secreted by osteoblasts, macrophage colony‐stimulating factor (M‐CSF) and RANKL, are responsible for osteoclast precursors proliferation and osteoclastogenesis. Importantly, RANKL could prevent apoptosis of osteoclasts^3^, ^4^ and induce expression of osteoclast‐specific markers and transcription factors such as nuclear factor of activated T cells c1(*NFATc1*).^4^, ^6^ As bone‐resorbing cells,^192^, ^193^, ^194^, ^195^ osteoclasts highly express bone resorption‐associated proteins including osteoclast‐specific markers cathepsin K (CTSK), tartrate resistant acid phosphatase (TRAP) and matrix metalloproteinase 9 (MMP‐9).^4^, ^196^ Specifically, CTSK breaks down organic components in bone,^197^, ^198^ TRAP is implicated in cell adhesion upon activation by CTSK,^199^, ^200^ and high levels of MMP‐9 commonly occur in resorption lacunae.^201^ Multiple fatty acids have been found to promote or suppress osteoclast activity, in most cases via regulation of RANKL signalling. Effects of fatty acids on osteoclast functions demonstrate their potential applications as therapeutic reagents against resorption‐associated bone disorders such as osteoporosis and rheumatoid arthritis.

#### 4.2.1 Fatty acids as positive regulators of osteoclasts

Accumulating evidence has shown that PA enhances RANKL‐mediated differentiation of osteoclasts by upregulating expression levels of RANK; importantly, PA has been reported to be sufficient for osteoclast differentiation in conditions even without RANKL.^202^

#### 4.2.2 Fatty acids as negative regulators of osteoclasts

##### LCPUFAs

LCPUFAs such as DHA and AA could exert inhibitory effects on osteoclast proliferation, differentiation and maturation. In mechanism, DHA intervention could inhibit osteoclast precursors proliferation by inhibiting M‐CSF‐induced activation of AKT and expression of cyclin D1/D2, and DHA triggers apoptosis of mature osteoclasts by inducing Bim expression and thus leads to defective osteoclast formation.^203^ In addition, DHA and AA could regulate migration and adhesion of osteoclasts in bone by downregulating expression of RANK and VNR.^204^ As for osteoclastogenesis process, LCPUFAs including DHA and AA suppress the expression of osteoclast‐specific genes such as *CTSK, TRAP, MMP‐9, NFATc1, c‐Fos* and *DC‐STAMP* in differentiating osteoclasts, thus decreasing osteoclast numbers and bone resorption.^110^, ^205^, ^206^, ^207^ In detail, DHA and AA bind to TLR4 on cell membrane to suppress TLR4 signalling, MAPK pathways and NF‐κB signalling.^208^ This further leads to downregulation of c‐Fos^205^ and NFATc1,^209^ which is the master regulator for osteoclast proliferation and differentiation. Also, levels of key cell‐to‐cell fusion mediator DC‐STAMP^179^, ^210^ are decreased, followed by substantial reduction in osteoclast formation and osteoclast number. Of note, there are certain differences between DHA and AA in combating bone resorption. Specifically, AA displays a more profound effect than DHA in inhibiting osteoclast function at equal concentrations,^206^, ^207^ which probably results from a more significant inhibition of CA2 expression^205^ and further prevention of resorption lacunae acidification with facilitation of CTSK and MMP‐9 enzymatic activities.^205^ These findings provide molecular mechanisms underlying the benefits of DHA supplement, and intake of high doses of EPA and DHA supplements has been suggested to attenuate bone loss associated with breast cancer.^211^

And, ALA intervention leads to apoptosis reactivation and RANKL signalling repression in osteoclasts. In mechanism, ALA reduces RANKL‐stimulated phosphorylation of JNK, ERK and AKT together with NF‐κB and BCL‐2 proteins to exert pro‐apoptotic action,^212^ reduces inflammatory bone loss via downregulating NF‐kB‐iNOS‐COX‐2 signalling axis and further inhibits RANKL‐induced osteoclast differentiation. Moreover, ALA can be converted into downstream fatty acids and several eicosanoids such as DHA and EPA and further exerts more complicated effects on osteoclastogenesis.^181^

##### LCMUFAs

Studies have shown that MA could suppress N‐myristoyl‐transferase, a critical enzyme involved in Src myristicylation^26^ to endoplasmic membrane and further phosphorylation.^25^ MA‐induced Src inhibition then affects a large number of cytoskeletal changes in osteoclasts, reduces latter stages of osteoclast differentiation and prevents RANKL‐induced bone loss in vivo.^25^ Such inhibitory effects against osteoclast formation and function suggest MA might serve as a new therapeutic agent against osteolytic bone disorders. In addition, investigations by Heerden *et al* have suggested that PLA could inhibit RANKL‐induced osteoclast formation and promote apoptosis of mature osteoclasts.^166^ In mechanism, PLA downregulates the activity of NF‐κB, MAPKs, JNK and ERK, inhibits expression of genes involved in osteoclast activity such as DC‐STAMP and resorption markers CTSK, MMP9 and TRAP and reduces number of TRAP‐positive osteoclasts by repressing actin ring formation and blocking their osteolytic capability,^166^ suggesting PLA as a potential therapeutic option for bone disorders related to excessive osteoclast formation. Moreover, as stated previously, PA enhances RANKL‐mediated osteoclastogenesis by facilitating expression of TNF‐α and RANK; conversely, OA could increase expression of DGAT1 and intracellular accumulation of triglycerides in osteoclasts to attenuate PA‐induced osteoclastogenesis.^202^ In addition, OA might facilitate osteogenic differentiation of adipose tissue‐derived stromal cells^168^ and thus serve as potential bone induction agent.

##### SCFAs

SCFAs represent useful supplements to inhibit bone resorption and restore bone metabolism balance.^64^ Among SCFAs, although concentrations of these molecules in vivo are too low to affect bone metabolism, in vitro investigations suggest that butyrate and propionate alone or mixed could inhibit osteoclast differentiation.^28^ In mechanism, SCFAs exert effects on bone metabolism (mainly inhibit bone resorption) via direct^30^or indirect^64^ mechanisms. Directly, SCFAs bind to receptors (GPR18, GPR41, GPR43, GPR109A)^30^ present on osteoclast precursors; in specific, acetate and propionate show higher affinity for GPR41, while butyrate exerts effects mostly via GPR43 activation.^30^ Indirectly,^64^ SCFAs regulate bone mineral absorption by influencing signalling pathways and gene expression. Butyrate and propionate induce metabolic reprogramming of osteoclasts to enhance glycolysis and thus downregulating critical genes in osteoclasts such as *TRAF6 and NFATc1*.^29^ In addition, production of SCFAs increases serum IGF‐1 and peripheral serotonin levels, which affects bone metabolism and decreases PTH levels to inhibit bone resorption.^28^, ^63^, ^213^ And, SCFAs might play a role in immunoregulation by modulating inflammatory events to prevent inflammatory bone loss such as arthritis.

##### MCFAs

CA inhibits RANKL‐modulated osteoclastogenesis in bone marrow‐derived macrophages by preventing M‐CSF and RANKL‐induced cytoskeletal reorganization, suppresses RANKL‐stimulated IκBα phosphorylation and enhanced NF‐κB transcription and diminishes RANKL‐induced NFATc1 activation.^214^ Moreover, CA could promote apoptosis of mature osteoclasts by initiating Bim expression and inhibiting M‐CSF‐induced ERK activation,^214^ demonstrating CA treatment represents a potential strategy for amelioration of bone resorption‐associated diseases.^214^

##### Fatty acids derivatives

As a metabolite of AA generated by lipoxidase, LXA_4_ could dose‐dependently reduce levels of ROS, the expression of osteoclast‐specific genes and osteoclast‐related transcription factors and thus attenuate osteoclasts‐mediated bone loss.^215^ And exposure to EPA‐derived RvE1 could downregulate STAT1 and subsequently attenuate MAPK and NF‐κB signalling,^152^ which further restore favourable receptor inducer for RANKL/OPG ratio and rescue OPG production, thus regulating osteoclast differentiation.^151^ Moreover, PGE2 could induce activation of osteoclasts in a dose‐dependent manner. In vivo studies have revealed that continuous treatment of rats with PGE2 results in bone loss owing to increased osteoclasts stimulation, and higher rates of bone resorption compared with bone formation due to longer bone resorption period.^188^ In vitro, PGE2 has been shown to increase osteoclast size, enhance resorptive pit formation and reduce osteoclast apoptosis.^216^ In mechanism, PGE2 could trigger osteoclastogenesis in murine bone marrow cultures treated with RANKL and M‐CSF, possibly caused by EP2 and EP4 receptor‐modulated induction of adenylate cyclase,^191^, ^217^ and by suppression of OPG and osteoblast‐induced RANKL secretion and enhanced RANK expression in osteoclasts.^218^, ^219^

### 4.3 Fatty acids and BMMSCs

BMMSCs are multipotent cells characterized by surface markers of CD105, CD73, CD90, CD44, CD29 and CD146^9^ with differential potentials into osteoblasts, chondroblasts and bone marrow adipocytes.^7^ BMMSCs are critical in maintaining the dynamic homeostasis of bone tissue, and deficiencies of BMMSCs proliferation are correlated with reduced bone mass.^220^, ^221^ Various signalling pathways including Wnt, Notch, Hedgehog, TGF‐β and BMP are involved in BMMSCs osteogenesis. Notably, Runx2 plays the most pivotal role in this process by promoting expression of osteogenesis‐related genes, regulating cell cycle progression and improving bone microenvironments.^8^

#### 4.3.1 Fatty acids as positive activators of BMMSCs

DHA, a special lipid component of osteoblast membrane, has been reported to fuel wide lipidomic remodelling of BMMSCs. DHA supplementation enhances Akt activation at plasma membrane and thereby potentiates osteogenic differentiation.^222^ Long‐term and high‐dose treatment of inflammatory diseases with Dex facilitates apoptosis of BMMSCs, leading to bone loss and associated metabolic bone diseases.^223^, ^224^ These effects can be eliminated by EPA via activating autophagy and suppressing apoptosis of BMMSCs. More specifically in the case of Dex‐induced apoptosis, activation of GPR120 by EPA triggers Ras‐Erk1/2 cascade, leading to suppression of Dex‐induced apoptosis, accompanied by activation of AMPK/mTOR to initiate autophagy.^223^, ^224^ Interestingly, EPA treatment in the absence of Dex has limited effects on autophagy induction,^223^, ^224^ demonstrating potential therapeutic role of EPA in managing long‐term side effects of Dex abuse.^225^

Oleate inhibits palmitate (palm)‐induced apoptosis and increases BMMSCs proliferation.^27^ Palm has been shown to induce lipotoxicity, whereas oleate fully neutralizes palm‐induced lipotoxicity and pro‐inflammatory response. Oleate exhibits cytoprotective effects by deactivating palm‐induced pathways and fostering esterification of Palm into triglycerides.^226^ More specifically, Ole inhibits palm‐induced activation of ERK and NF‐κB signalling, which results in pro‐apoptotic effects in BMMSCs.^226^, ^227^ Also, decline in IL‐6 and IL‐8 expression and secretion levels by Ole treatment was also observed.^228^ Furthermore, Ole maintains the oxidative levels of palmitate.^27^ Hence, OA represents a potential therapeutic agent in combating PA‐induced lipotoxicity in the bone.

#### 4.3.2 Fatty acids as negative regulators of BMMSCs

As mentioned above, palmitate triggers BMMSCs apoptosis and reduces their proliferation.^27^ Gillet *et al* have reported that palmitate exerts cytotoxic effects by inducing endoplasmic reticulum stress and activating NF‐κB and ERK signalling pathways, thus further regulating secretion of cytokines and chemokines in BMMSCs and inducing binding of exogenous ligands to TLRs. Moreover, palmitate triggers pro‐inflammatory responses *via* upregulating TLR4 expression accompanied with enhanced expression and secretion of IL‐6 and IL‐8, whose overproduction facilitates differentiation of osteoclast precursor cells into mature osteoclasts and results in impaired bone formation and enhanced bone resorption.^229^, ^230^, ^231^, ^232^ And undifferentiated BMMSCs have been found to be less sensitive to lipotoxicity than BMMSC‐derived osteoblastic cells.^226^

### 4.4 Fatty acids and osteocytes

Osteocytes are osteoblast‐derived cells located in lacunae surrounded by mineralized bone matrix, with the ability to support bone structure and receive machine sensation. Importantly, osteocytes can serve as endocrine cells to synthesize and express important regulatory molecules including RANKL, Dickkopf‐1 (DKK1) and sclerostin (SOST)^233^, ^234^, ^235^ and thus participating in bone resorption and formation regulation by coupling osteoclast and osteoblast activities.^6^ Studies have shown that fatty acids such as PA and PGE2 have noteworthy influences on osteocyte metabolism, which might provide novel therapeutic strategies for bone diseases like osteoporosis.

#### 4.4.1 Fatty acids in osteocytes‐mediated bone metabolism

PGE2 released by osteocytes are important regulators of bone formation. For example, PGE2 produced by low‐intensity pulsed ultrasound‐stimulated osteocytes could enhance osteoblasts differentiation but inhibit their proliferation in vitro.^236^ In addition, mechanical loading or fluid flow shear stress on osteocytes can release PGE2 to regulate osteoblast proliferation and differentiation.^237^ In mechanism, loading‐induced PGE2 can activate EP2/EP4 receptors to stimulate downstream PI3K/Akt pathway,^238^ which further facilitates gap junction communication by transcriptional regulation of Cx43 to promote osteocytes survival.^239^ And PGE2 can activate MAPK and subsequently induce phosphorylation of Cx43 at S279/282 and closure of Cx43 hemichannels, which thus modulating bone anabolism and protecting osteocytes from harmful effects caused by sustained hemichannels opening.^239^ Moreover, PGE2 could promote production of 8‐nitro‐cGMP in osteocytes to enhance osteoclasts differentiation.^240^

#### 4.4.2 Fatty acids in osteocytes‐associated bone disorders

Investigations have suggested that PA can cause lipotoxicity in osteocytes. PA results in apoptosis and inhibits survival in osteocytes by induction of autophagy failure, which is indicated by conspicuous increase in LC3‐II and reduction of autophagosomes/lysosomes in cytoplasm.^234^ In addition, PA exerts effects on bone turnover by decreasing expression of DKK1, RANKL and sclerostin in osteocytes.^234^ Given osteocytes apoptosis and dysfunction are two common changes in osteoporotic bone, PA might play a part in the pathogenesis as well as potential therapeutic applications in osteoporosis. In addition, fatty acids oxidation can serve as energy source for osteocytes.^241^ In vivo evidence has shown that fatty acid oxidation could compensate dysfunction of energy metabolism and osteocytes formation caused by glucose transporter‐4 deficiency in osteoblasts and osteocytes of mice.^242^ Importantly, activation of β‐catenin regulated by Wnt‐Lrp5 signalling affects oxidative potential and fatty acids utilization in osteocytes and thus is responsible for expression of key enzymes during fatty acid oxidation.^241^ Therefore, fatty acid oxidation in osteocytes exerts regulatory effects on bone fat and body mass, which might have regulatory roles and therapeutic applications in metabolic disease‐associated bone disorders.

### 4.5 Fatty acids and chondrocytes

Chondrocytes is the main cartilage cell type existing in cartilaginous interstitium and cartilage lacuna, and they can produce cartilage extra cellular matrix that composed mainly of proteoglycans and collagen.^243^ Fatty acids are integrated into chondrocytes mainly in the form of phosphatidylcholine and triacylglycerols and then mediate downstream signalling pathways via receptors expressed on chondrocytes membrane such as GPR40, GPR120, CD36 and TLR4, as well as a few LRP and PPAR family members.^12^, ^244^ As an energy source for chondrocytes, fatty acids participate in chondrocytes energy metabolism^245^ and further alleviate or enhance chondrocytes damage and cartilage degeneration *via* multiple mechanisms. Understanding the regulation effects of fatty acids in chondrocytes might help to explore their potential therapeutic values for bone disorders associated with chondrocytes inflammation and cartilage degeneration.

#### 4.5.1 Fatty acids as positive regulators of chondrocytes

##### ω‐3 PUFAs and metabolites

EPA plays anti‐inflammatory roles by competitively suppressing AA oxidation pathway,^246^ and EPA treatment could delay IL‐α‐induced chondrocyte death.^247^ In addition, EPA treatment could inhibit oxidative stress‐induced chondrocyte apoptosis *via* poly (ADP‐ribose) polymerase and caspase 3 cleavage, p38 MAPK, p53 phosphorylation and MMPs expression and thus ameliorating cartilage degeneration.^248^ p38 MAPK‐dependent mechanism is also involved in DHA‐involved alleviation of cartilage damage.^249^

EPA and DHA can be converted to SPM and novel bioactive lipid mediators such as resolvins in vivo.^250^ Articular chondrocytes could participate in SPM metabolism by expressing biosynthetic enzymes like15‐LO type 1,^251^ and SPM exhibits a more potent anti‐inflammatory effect than their precursors in protecting chondrocytes and cartilage.^12^, ^250^ As for resolvins, resolvin D1 demonstrated anti‐arthritic nature in a model of inflammatory arthritis indicated by significantly attenuated arthritic score and hind paw oedema and reduced leucocytes infiltration within paw.^252^

Resolvin D3 also shows similar effect on arthritis model.^253^ In mechanism, investigations by Benabdoune *et al* in an experimental osteoarthritis in human chondrocytes have found that RvD1 inhibits IL‐1β‐induced COX2, PGE2, inducible NO and MMP‐13 by stifling IL‐1β‐induced activation of p38/MAPK, JNK1/2 and NF‐κB/p65.^254^ Moreover, resolvin D1 could maintain cartilage integrity in inflammatory arthritis by stimulating the production of chondrocytes extracellular matrix and inhibiting IL‐1β‐induced cells degradation *via* direct ALX/FPR2 receptor ligation.^252^ And, resolvin D1 could reduce 4‐hydroxynonenal‐induced oxidative stress and chondrocytes apoptosis.^254^ These findings suggest that it is promising to develop novel therapeutic strategies based on the functional mechanisms of SPM for the therapeutics of chondrocyte‐related diseases such as osteoarthritis.^251^

##### AA derivatives

As epoxide metabolites of AA, epoxyeicosatrienoic acids (EETs) have been reported to reduce inflammatory cytokines such as TNF‐α and IL‐6 and decrease cytotoxicity in canine chondrocytes. However, since EETs could be rapidly metabolized into corresponding vicinal diols by soluble epoxide hydrolase (sEH), sEH inhibitors that are able to stabilize anti‐inflammatory EETs might have therapeutic potentials for chondrocytes survival and cartilage protection.^255^

##### SCFAs

Butyric acid and butyrate can reduce cartilage destruction mainly by inhibiting inflammation and MMPs expression. Studies have shown that in human chondrocytes, butyric acid could dose‐dependently suppress IL‐1β‐induced PGE2 synthesis as well as TNF‐α/IL‐17‐induced PGE2 production, with a mechanism involving COX‐2 expression inhibition.^256^ And butyric acid can reduce the release of IL‐1β‐induced proteoglycan from cartilage explants.^256^ Butyrate could inhibit the production of key MMPs in chondrocytes via pro‐inflammatory cytokines at both mRNA and protein levels, which further potently inhibit cartilage collagen breakdown.^257^ Moreover, sodium butyrate markedly inhibits IL‐1β‐induced expression of MMPs and ADAMTSs by suppressing phosphorylation of IκBα, NF‐κB p65 and IKK to abolish inflammatory NF‐κB activation.^258^ Importantly, GPR43 receptor is greatly relevant to efficacy of butyrate in inhibiting IL‐1β‐induced inflammation in chondrocytes and its chemoattractant effects.^259^

#### 4.5.2 Fatty acids as negative regulators of chondrocytes

##### SFA and its metabolites

Several studies have shown that animals fed with high‐SFAs diet exhibit accelerated cartilage degeneration,^260^ and long‐chain SFAs are considered as important negative regulators of chondrocyte metabolism. Studies have shown that BMMSCs and adipose stem cells‐derived chondrocytes which generate long‐chain SFAs have decreased cartilaginous matrix production,^261^ and SFAs with different chain lengths might exert relative effects in chondrocytes. It has been found that diet rich in longer chain SFAs such as PA and SA promotes more expression of collagenase‐10 and MMP‐13 and increases much more chondrocyte apoptosis than diet rich in shorter chain SFAs.^260^

PA and SA have been reported to participate in inflammatory reactions by augmenting pro‐inflammatory markers such as IL‐6 in human chondrocytes.^262^ In primary mouse chondrocytes, SA could promote lactate dehydrogenase‐dependent production of lactate to stabilize HIF1α protein and facilitate pro‐inflammatory cytokines expression^263^ SA‐stimulated NF‐κβ p65 activation and pro‐inflammatory cytokines expression in chondrocytes could be attenuated by miRNA‐26a; conversely, NF‐κB p65 could also inhibit miRNA‐26a production by directly targeting the promoter region of miRNA‐26a.^264^ In addition, PA and SA treatment could enhance autophagy activation in chondrocytes, which is strongly associated with increased activation of NF‐κB signalling pathway,^265^ while opposite effects have been observed upon LA stimulation.

Palmitate has been reported to synergize with IL‐1β to induce caspase activation and chondrocyte apoptosis, as well as increase expression of cyclooxygenase 2 and IL‐6 in chondrocytes via TLR‐4 signalling, which are all involved in the pathological processes of cartilage destruction.^266^, ^267^ Lipotoxicity of palmitate could also be mediated by endoplasmic reticulum (ER) stress and further suppresses IGF‐1‐mediated signalling and succedent proteoglycans and collagen type II synthesis in chondrocytes.^268^ And utilization of either JNK inhibitor or small molecule chemical chaperone could weaken the effect of palmitate to facilitate cartilage matrix synthesis and chondrocytes survival.^269^, ^270^ Importantly, palmitate‐induced ER stress could activate unfolded protein response signalling and subsequently promote apoptosis of meniscus cells to affect the development of obesity‐related osteoarthritis.^271^ Moreover, in human chondrocytes, palmitate could induce expression of pro‐apoptotic molecules such as cleaved caspase‐3 (CC3) and negative cell survival regulators such as tribbles related protein 3 (TRB3) and nuclear protein 1 (Nupr1) and thus induces apoptosis of chondrocytes.^272^ Taken together, palmitate has potent therapeutic implications for inflammatory bone diseases such as osteoarthritis.

##### ω‐6 PUFAs and their metabolites

A growing body of evidence has shown that a higher ratio of ω‐6‐to‐ω‐3 PUFAs might exert negative influences on cartilage.^273^ As for specific mechanisms, ω‐6 PUFAs such as ALA and AA aggravate cartilage damage by serving as precursors for pro‐inflammatory prostanoids, while ω‐3 PUFAs such as EPA and DHA protect cartilage by being metabolized to anti‐inflammatory mediators such as protectins and resolvins.^273^, ^274^ Moreover, AA‐derived PGE2 could serve as important inflammatory mediator to regulate inflammatory reactions of chondrocytes. Studies have shown that PGE2 could suppress differentiation of chondrocytes by activating downstream receptors protein kinase A (PKA) and protein kinase C (PKC), which might be responsible for activation of transcription factors associated with collagen X production.^275^

**Figure 4:**
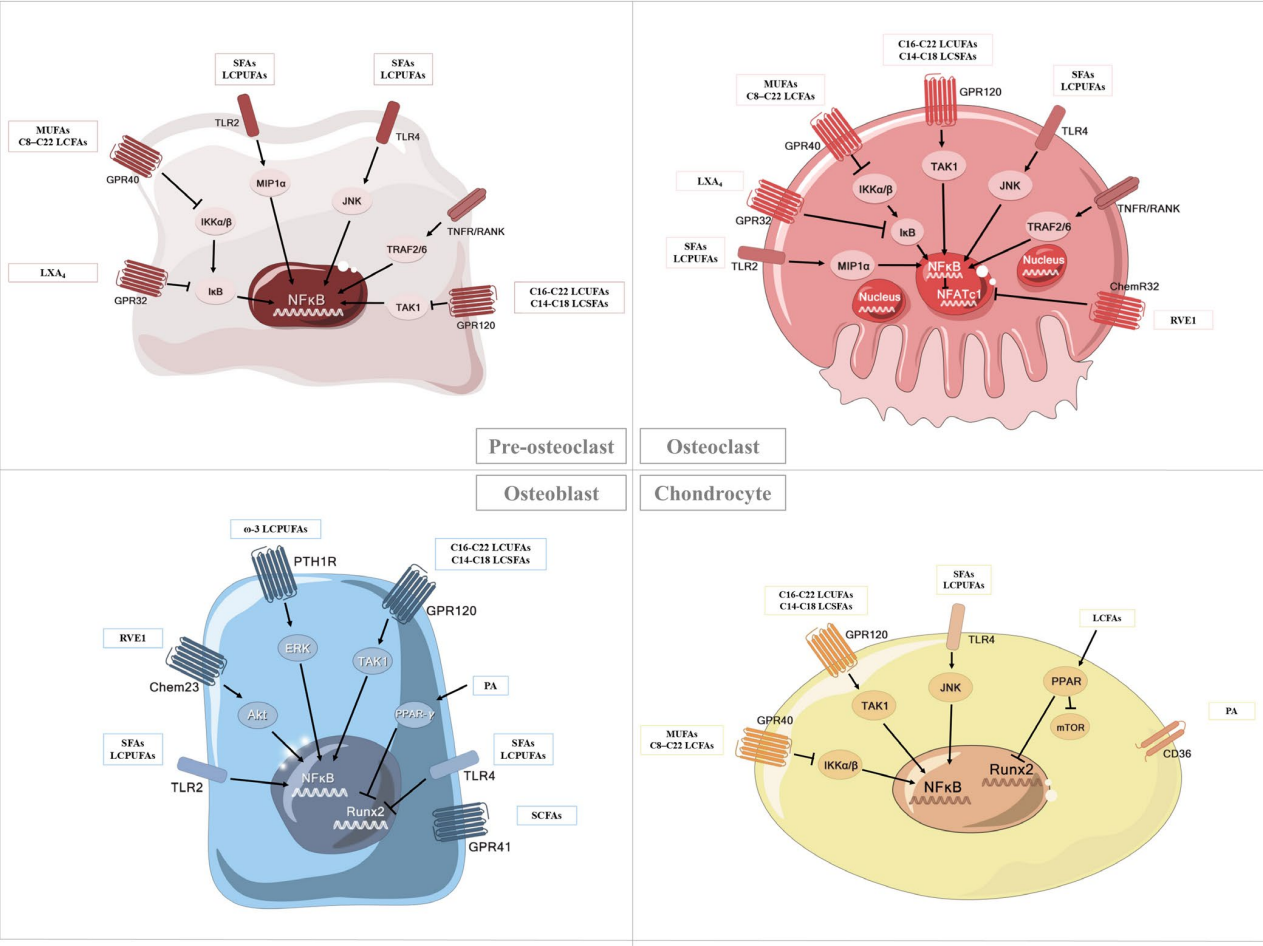
Modulation of fatty acids on specific bone cell types. Multiple receptors for fatty acids including GPRs, ChemR23, TLRs and PPARs are found in pre‐osteoclasts, mature osteoclasts, osteoblasts and chondrocytes. Several GPRs including GPR18, GPR41, GPR43 and GPR109A are receptors for SCFAs (C2‐C5) expressed in both osteoclasts and osteoblasts. GPR40, found on osteoclasts, could be activated by medium/long‐chain fatty acids with a chain length of C8‐C22. GPR84, whose expression in macrophages and adipocytes could be enhanced under inflammatory conditions, is a receptor for MCFAs (C9‐C14). GPR120 is expressed on osteoblasts and osteoclasts and could be stimulated by LCSFAs (C14‐C18) and LCUFAs (C16‐C22). PTH1R, belonging to GPR2 family, could be antagonized by ω‐3 LCPUFAs to promote osteoblast activity. PPARs, with known ligands including LCPUFAs and metabolites such as PGE2, are nuclear receptors that regulate lipid metabolism by acting as transcription factors in BMMSCs, osteoblasts, osteoclasts and chondrocytes. TLRs, including TLR2 and TLR4, are found in osteoblasts, pre‐osteoclasts, osteoclasts and chondrocytes. Their ligands are mainly SFAs and LCPUFAs and are involved in inflammatory action. ChemR23 can act as chemerin receptor as well as RvE1 receptor in bone tissue cells such as osteoclasts and osteoblasts. Interactions of fatty acids with specific receptors induce transduction of transmembrane specific signals and activation of various downstream signalling pathways including NF‐κB, NFATc1 or Runx2‐mediated transcriptional regulation, and further modulating bone microenvironment homeostasis and pathological bone remodelling. GPRs, G protein‐coupled receptors; chemR, chemokine‐like receptor; TLR, toll‐like receptor; SCFAs, short‐chain fatty acids; MCFAs, medium‐chain fatty acids; LCSFAs, long‐chain saturated fatty acids; LCUFAs, long‐chain unsaturated fatty acids; PTH1R, parathyroid hormone type 1 receptor; LCPUFAs, long‐chain polyunsaturated fatty acids; PPARs, peroxisome proliferator‐activated receptors; PGE2, prostaglandin E2; SFAs, saturated fatty acids; RvE1, resolvin E1; NF‐κB, nuclear factor‐kappa B; NFATc1, nuclear factor of activated T‐cell cytoplasmic 1; Runx2, runt‐related transcription factor 2

Taken together, fatty acids exert multiple effects on specific bone cell types and thereby associated bone diseases (Table 4), which might be mediated *via* distinct mechanisms at cellular and molecular levels (Figure 4). Understanding the mechanistic implications of fatty acids in bone cells will greatly benefit their further utilization in related bone disorders.

**Table 4:** Modulatory roles and therapeutic potentials of fatty acids for bone diseases

Disease	Pathologic mechanism	Fatty acid	Detrimental mechanism	Potential drug formula	Therapeutic mechanism	Reference
Periodontitis	Specific bacterial colonization Increased function of osteoclasts Increased dysfunction of osteoblasts	PA	Induces pro‐inflammatory response	ω‐3 LCPUFAs	Inhibit putative periodontal pathogens Inhibit PA‐induced chemokine secretion	31, 33, 49
		Butyrate	Inhibit differentiation of HGFs	Resolvin D1	Inhibit putative periodontal pathogens Inhibit PA‐induced chemokine secretion	
		SFAs	Induce oxidative stress Facilitate inflammatory processes	RvE1	Reduce inflammation Inhibit osteoclast activities	
Osteoporosis	Increased function of osteoclasts Increased dysfunction of osteoblasts	ω‐6 LCPUFAs	Induce chronic inflammation Induce MSC chronic deregulation	ω‐3 LCPUFAs	Inhibit osteoclastogenesis Reduce PGE2	51, 52, 58
				PA	Provide energy generation for differentiation of osteoblasts	
Osteoporosis	Reduced OPG Facilitated osteoclast differentiation	—	—	ω‐3 LCPUFAs	Inhibit osteoclastogenesis Reduce PGE2	47, 61, 64, 65
		—	—	SCFAs	Inhibit osteoclast differentiation Provide energy generation for differentiation of osteoblasts	
Bone fracture	Deterioration of bone structure Loss of bone mineral	ω‐6 LCPUFAs	Induce PGE2 production	ω‐3 LCPUFAs	Increase calcium resorption Increase synthesis of bone collagen Inhibit urinary calcium excretion	67, 68
Rheumatoid arthritis	Autoimmune inflammatory disease of unknown aetiology	ω‐6 LCPUFAs	Induce production of pro‐inflammatory cytokines	ω‐3 LCPUFAs	Reduce inflammation Reduce cartilage‐degrading enzymes	75, 76
Osteocarcinoma	Derives from primary bone sarcomas or prostate cancer, breast cancer etc	AA	Supports implantation and propagation of metastatic cells	DHA	Reduce CD44 expression in metastatic cells Inhibit osteoclast formation	88, 89, 90, 91, 92, 94, 95, 96
				EPA	Reduce CD44 expression in metastatic cells	
Osteomyelitis	Bone infection of pyogenic organisms	—	—	ω‐3 LCPUFAs	Reduce levels of TNF‐α and IL‐6 Reduce SOD activity	97, 101, 102, 103, 298
Multiple myeloma	Cancer growing in bone marrow	SFAs	—	PA	Activate multiple myeloma cell apoptosis	94, 95, 96, 97, 98, 99, 100, 101
		ω‐6 LCPUFAs	—	ω‐3 LCPUFAs	Promote drug sensitivity of myeloma cell apoptosis Activate multiple myeloma cell apoptosis Inhibit function of ω‐6 LCPUFAs	

## 5 CONCLUDING REMARKS

In this review, we reviewed impacts of fatty acids on bone metabolism, summarized molecular mechanisms involved in actions of fatty acids in distinct bone cell types, and discussed their potential implications for metabolic bone disorders. Currently available findings imply that LCPUFAs mainly exert protective functions on bone by promoting functions of BMMSCs and osteoblasts while inhibiting activities of osteoclasts. MCFAs such as CA suppress osteoclastogenesis and thereby alleviate bone resorption. SCFAs and associated combinational treatment might inhibit bone resorption and inflammatory response for potential therapeutics against inflammatory bone loss including arthritis. Overall, these fatty acids might serve as potential therapeutic and nutritional agents in managing metabolic bone disorders such as osteoporosis, rheumatoid arthritis and oral‐maxillofacial diseases such as periodontitis. Moreover, as natural compounds occurring widely in human body, fatty acids are available in a variety of ways and might be potent to antagonize possible side effects of current drug therapies. Nevertheless, currently available investigations have only reported roles of fatty acids in a limited number of bone disorder conditions, and further bench and clinical investigations are needed to comprehensively elucidate the underlying mechanisms for their possible applications in additional skeletal disorders such as temporomandibular joint disorder and osteosarcoma. Taken together, we conclude that involvement of fatty acids in bone diseases pathogenesis might provide potential therapeutic targets for interventions of bone disorders, and promising fatty acids with therapeutic effects might be used directly or indirectly in nutritional or drug formulations for prevention and treatment of specific types of bone disorders.

## CONFLICT OF INTERESTS

The authors declare no conflicts of interest.

## AUTHOR CONTRIBUTIONS

Bao M and Zhang K gathered relevant literature and wrote the manuscript; Wei Y, Hua W and Gao Y interpreted data from pathological and experimental studies; and Li X and Ye L provided financial support, revised and reviewed the manuscript.

## Data Availability

The data that support the findings of this study are available from the corresponding author upon reasonable request.
